# A preliminary study of motivational interviewing as a prelude to intensive treatment for an eating disorder

**DOI:** 10.1186/2050-2974-1-34

**Published:** 2013-08-20

**Authors:** Carmen V Weiss, Jennifer S Mills, Henny A Westra, Jacqueline C Carter

**Affiliations:** 1 Department of Psychiatry, St. Joseph’s Healthcare, Hamilton, Ontario, Canada; 2 Department of Psychology, York University, Toronto, Ontario, Canada; 3 Department of Psychology, Memorial University of Newfoundland, St. John’s, Newfoundland, Canada

**Keywords:** Motivational interviewing, Eating disorders, Treatment

## Abstract

**Background:**

Engaging patients with an eating disorder in change is difficult and intensive treatment programs have high drop-out rates. The purpose of the study was to determine whether Motivational Interviewing (MI) in the form of a brief, pre-treatment intervention would be associated with higher completion rates in subsequent intensive treatment for an eating disorder.

Thirty-two participants diagnosed with an eating disorder participated in the study. All participants were on the waitlist for admission to an intensive, hospital-based treatment program. Sixteen participants were randomly assigned to four individual sessions of MI that began prior to entrance into the treatment program (MI condition) and 16 participants were assigned to treatment as usual (control condition). The main outcome was completion of the intensive treatment program. Participants also completed self-report measures of motivation to change.

**Results:**

Participants in the MI condition were significantly more likely to complete intensive treatment (69% completion rate) than were those in the control condition (31%).

**Conclusions:**

MI can be a useful intervention to engage individuals with severe eating disorders prior to participation in intensive treatment. MI as a brief prelude to hospital-based treatment for an eating disorder may help to improve completion rates in such programs. Further research is required to determine the precise therapeutic mechanisms of change in MI.

## Background

Motivational Interviewing (MI) [1] is a treatment approach that was originally designed for use within the field of addictions to help enhance motivation to recover from substance abuse. In general, MI seeks to explore and resolve a client’s ambivalence toward change by acknowledging both the pros and cons of changing behaviour, by normalizing the experience of ambivalence, and by helping the client situate his/her behaviour within the context of his/her values and goals [1]. Resistance to change in a therapeutic context is seen as an indication of a mismatch between the intervention and the client’s readiness to change. Such resistance is considered to be a warning to the therapist to begin to validate the client’s concerns about making changes, rather than pushing for behavioural change, which would likely be met with more resistance. In so doing, the therapist gives the client the opportunity to consider and then critically evaluate both the benefits and costs of change. MI facilitates a movement towards change by encouraging the client to put the pros and cons in the context of his/her values and goals. Originally developed for patients with substance abuse, MI is generally well suited for use as an intervention for conditions in which ambivalence is common.

There is agreement among clinicians that eating disorders, including anorexia nervosa (AN) and bulimia nervosa (BN) are very difficult disorders to treat. Treatment for these disorders is often marked by premature drop-out and relapse [2]. Intensive, hospital-based treatment programs for eating disorders have notoriously poor completion rates and high rates of recidivism. Ambivalence toward recovery and low motivation to change are hallmarks of eating disorders [3]. Motivation to change has been found to be especially low in individuals with AN [4-6]. Other studies have reported no differences between these two groups on motivation to change [7,8].

Understanding ED patients’ ambivalence toward recovery and increasing their motivation to change is critical to increasing treatment effectiveness for these disorders. Early research by Geller and colleagues was among the first of studies to show a link between readiness to change one’s eating and outcome from treatment for an eating disorder [9]. A study by Bewell and Carter sought to determine whether a patient’s ambivalence about recovery would predict completion of an intensive hospital-based treatment program for AN [10]. They found not only that readiness to change eating and weight, as measured after four weeks in the program, predicted intensive treatment completion, but also that readiness to change fully mediated the relationship between ED symptomatology at admission to the program and later treatment completion. Similar findings were demonstrated in an adolescent population treated in an intensive hospital-based treatment program [11]. Other research has shown than individuals with BN who are more motivated to change at baseline experience a greater reduction in binge eating during therapy than do those who are initially less motivated to change [12,13], and that motivation to change has been shown to be a predictor of relapse in individuals with BN [14,15]. In sum, there is strong evidence that readiness for change impacts the course of treatment for an eating disorder.

Because MI specifically targets ambivalence about change, it has been thought to be useful in the treatment of eating disorders. Some studies have shown that MI and MI-based treatments are useful as either a stand-alone treatment or as a pre-treatment in recovery from binge eating [16,17]. There has also been some evidence that MI-based treatments and assessment techniques have been associated with increases in motivation and readiness to change in individuals with AN [18,19]. On the other hand, recent reviews by Dray and Wade [20] as well as Knowles and colleagues [21] conclude that although MI approaches appear to increase motivation in participants, there is still no compelling scientific evidence that MI approaches actually enhance treatment outcomes for eating disorders. Better designed studies, including the use of randomized controlled trials and improved treatment integrity, are called for.

Individuals with an eating disorder who enter and then prematurely drop out of intensive treatment do so for various reasons, including inability and unwillingness to comply with program rules (e.g., abstention from symptoms) or distress from eating that is so significant as to interfere with treatment. Given the substantial financial cost of these programs and the paucity of admission spots that are available for eating disorders, research needs to uncover ways in which to make individuals more likely to successfully complete treatment. The extant literature on motivation to change eating disorder symptoms suggests that it may be important to consider (and foster) an individual’s level of motivation to change before engaging the individual in intensive treatment for disordered eating. The present research evaluated the clinical efficacy of a brief MI intervention delivered to patients before admission to an intensive treatment program for an eating disorder. Based on the previous literature, it was hypothesized that individuals in the MI treatment condition would be more likely to complete the intensive treatment program than were those who did not receive the MI pre-treatment. Specifically, Gowers and Smyth [22] found that an MI pre-treatment led to improvements in self-reported motivation to change, which then predicted early treatment response. Therefore, it was further hypothesized that participants in the MI treatment condition would show a larger increase in their motivation to change (as measured by self-report questionnaires) across the 4-week pre-treatment interval than would those in the control condition.

## Methods

### Participants

The participants were recruited from the waiting lists for the Inpatient and Day Hospital Units of Toronto General Hospital’s Eating Disorder Program between January 2008 and June 2009. The inpatient and ambulatory programs for eating disorders at this hospital have been described in detail elsewhere [23]. Briefly, they are both well-established intensive group therapy programs that treat adult patients (ages 17 and older) and are primarily directed towards symptom control, normalized eating, and body weight restoration to a minimum body mass index (BMI) of 20 (in the case of those who are initially underweight). The inpatient program is typically recommended for patients who are potentially medically unstable and is primarily comprised of patients with AN. Patients begin the program as inpatients and are granted outside privileges as they demonstrate symptom control and meet minimal standards of weight gain. Approximately two thirds of the way through their stay, if they are progressing well in their recovery, they are discharged as an inpatient, but continue to attend the program as a day-patient (i.e., attending all groups and eating two meals and a snack with the inpatient group). Once patients reach a BMI of 20, they typically are kept in the program for 2–3 additional weeks, to solidify their gains, and then they are discharged to the follow-up care program at the hospital. The ambulatory treatment program is comprised entirely of outpatients. However, the program is intensive, as patients typically attend the program from 10 am until 6 pm, Monday to Friday, and complete two staff-supervised meals and one supervised snack each day in hospital. The ambulatory program is comprised of individuals with clinical and subclinical levels of AN and BN. Patients who do not require weight gain are given a maximum admission of eight weeks, whereas those who have weight to gain are admitted until they reach a BMI of 20.

All participants in this study were female and met DSM-IV criteria for AN, BN, or Eating Disorder Not Otherwise Specified (EDNOS). These assessments were completed by psychologists, psychiatrists, and Master’s level therapists in the Toronto General Hospital Eating Disorders program. Participants also were required to have a BMI greater than or equal to 13, as we were concerned that patients with a BMI of less than 13 would be too medically unstable to participate. It was decided that clients who were suicidal (i.e., those who expressed suicidal intent and plans for how they would hurt themselves) would be included in the study since suicidality is so common in eating disordered populations [24]. These patients were monitored closely throughout pre-treatment and intensive treatment. Only one patient demonstrated severe suicidal ideation, and she was admitted to an acute mental health unit after only one MI session, and was subsequently excluded from the analyses.

**Figure 1:**
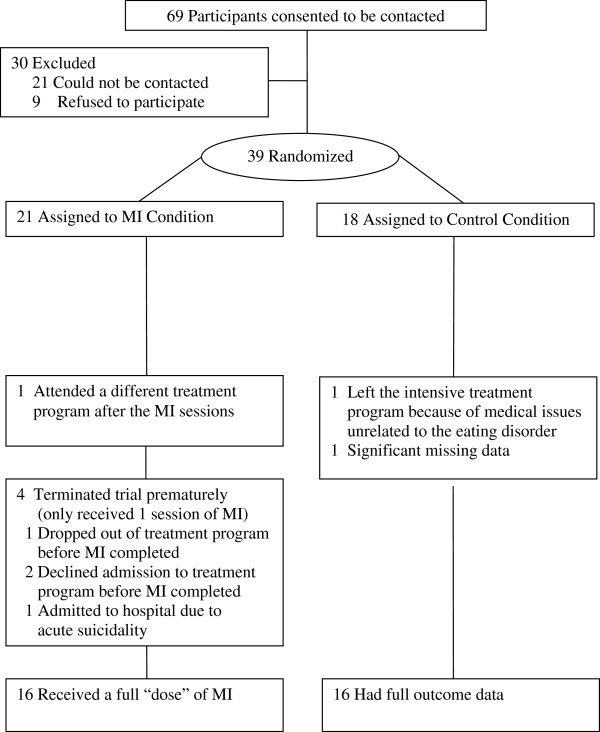
Flow of participants through the study.

In total, 69 participants meeting the inclusion criteria agreed to be contacted about the study (see Figure 1). Of these individuals, 39 (56.5%) consented to participate and entered the trial. One participant in the MI condition was excluded because she attended a different hospital’s treatment program after completing her MI sessions.^a^ Two participants in the control condition were excluded; one had a disruption in her intensive treatment due to unrelated medical issues and one had significant missing questionnaire data.

### Definition of treatment completion

Twenty-one participants were randomized to the MI condition. Four patients completed only one session, two completed three sessions, and 14 completed all four MI sessions. There are currently no guidelines for an appropriate “dose” of MI, but it was agreed by the authors that completion of three out of four sessions of MI could be considered treatment completion. It was noted by the first author that there was little new material introduced after the third session and that the fourth session served mainly to reiterate issues raised in previous sessions. Before excluding participants on the basis of MI non-completion, a series of t-tests were run to compare the groups on all the measures of motivation measured at pre-randomization. It was determined that there was only one difference between the “completers” and the “non-completers”: the MI non-completers (i.e., the four participants who received only one out of four sessions) had lower baseline scores on confidence about changing weight and shape preoccupation (*t* (16) = 3.20, *p* = 0.01). There was no evidence that this variable predicted hospital treatment completion or introduced other confounds. Thus, it was decided that, with no other differences between the groups, the four participants who received only one session of MI would be excluded from further analysis.

The final sample consisted of 32 participants. Sixteen had been randomly assigned to the MI treatment condition (15 females, 1 male), and 16 to the waiting list control condition (15 females, 1 male) (see Figure 1).

### Procedure

This study received ethical approval from the Research Ethics Boards of both York University and the hospital ethics board. Participants who consented to participate were asked to complete some self-report questionnaires. They also participated in a brief assessment interview inquiring about demographic information, diagnostic information and age of onset of their eating disorder, eating disorder treatment history, and information about the treatment they were receiving at the time of randomization. Finally, participants were randomized (through permuted block randomization) into either the treatment or control condition. Treatment staff in the subsequent intensive treatment program was kept blind to participants’ group assignment.

#### Treatment condition

The MI condition received weekly 50-minute sessions of MI over four consecutive weeks. Treatment followed the principles and techniques outlined in Miller and Rollnick’s MI manual. At the end of each session, participants completed a measure of therapeutic alliance. It should be noted that we considered our MI intervention to be “brief” only in comparison to the typical length of treatment for an eating disorder. MI as an adjunct to other treatments is usually done over 1–2 one hour sessions. However, we wanted our intervention to include all of the key components to Miller and Rollnick’s MI manual. Participants in the MI condition were also able to continue to receive “treatment-as-usual,” meaning they could carry on as they normally would (e.g., seeing a family doctor, taking medication, etc.). This treatment most often included regular medical monitoring by their physician or psychiatrist and the use of anti-depressant medication. Some participants were also receiving psychotherapy with psychologists and clinical social workers, and some were seeing dietitians, as is common.

#### Control condition

Participants assigned to the control condition did not receive any MI treatment over the four-week treatment period, but remained on the waiting list and received treatment as usual. Admission to intensive treatment was not delayed for any participant as a function of condition.

Approximately four weeks after randomization (and after the final therapy session for those in the MI condition), participants in both groups were asked to complete another copy of the original questionnaire packet. They also consented to allow the researcher to access their hospital chart to determine whether they completed the subsequent treatment program, and these charts were examined to determine whether the participant completed treatment or dropped out prematurely.

The length of time between randomization and the participant’s admission to the intensive treatment program varied considerably (ranging from .43 to 30.5 weeks). This inconsistency was primarily accounted for by the varying nature of the waitlists for both of the hospital treatment programs. At the time of randomization, it wasn’t known when the participant would be entering intensive treatment. When the waitlists were short, there was often not sufficient time to recruit participants and finish the four-week assessment period before the participant was admitted to the program. Thus, 24 (75%) of participants were attending the treatment program for some portion of time while they were receiving the MI treatment or waiting to complete the final questionnaire packet (if they were in the control group). In other words, for some participants in both conditions, “treatment as usual” included intensive treatment in the hospital eating disorder program. A Chi-square analysis indicated that there were no differences between the MI group and the control group in the proportions of participants who completed their second set of questionnaires while in the program, *Χ*^*2*^(1, 32) = 3.46, *p* = .06.

### Therapist and therapist training

This study formed part of the doctoral dissertation of the first author (CW) and all MI treatment was delivered by this author. Training consisted of attending a 2-day intensive workshop on MI led by Dr. William Miller (one of the founders of MI), readings, on-going supervision (including live observation and videotape review) with a highly experienced MI therapist (HW), and on-going supervision with a highly experienced therapist specializing in eating disorders (JC).

### Measures

Pros and Cons of Eating Disorders Scale (PCED) [25]. This self-report measure asks participants to identify the extent to which they agree with 70 statements addressing both the pros and cons of ED. The items have been shown to have good internal consistency and test-retest reliability (ranging from .72 to .92) [26].

#### Motivation to change scale – modified version (MTC)

Motivation rulers (motivation, confidence, readiness) were used in the current study. These measures have been used previously in other research and have shown good psychometric properties [27], but have not yet been validated for use with eating disorders. Our measure was based on a scale comprised of three questions aimed at addressing an individual’s motivation to change his/her behaviour (“How important is it for you to eat normally and to gain weight?”), confidence (“If you decided to change your eating and weight, how confident are you that you would succeed?”), and feelings of readiness to change (“How ready are you to change your eating and weight?”). For clarity, the original questions were also changed by deleting any reference about gaining weight, because some of the patients had normal BMIs. In other words, “How important is it for you to eat normally and to gain weight?” became “How important is it for you to eat normally?” Each question is presented in a 10-point Likert-scale format. The current study also included questions re-worded for the symptoms of both Food Restriction and Weight/Shape Preoccupation.

### MI treatment integrity

Twenty percent of the MI treatment sessions were randomly selected and a twenty-minute videotaped segment was randomly chosen from each. These segments were then coded by two independent raters according to the Motivational Interviewing Treatment Integrity coding system (MITI, 2.0) [28]. These raters had been trained in the MITI rating system, and participated as raters on a previous MI treatment trial [29]. The MITI coding system creates global rating scores based on two dimensions: Empathy/Understanding and Spirit. Treatment integrity in the present study was set as 5 on a 7-point scale, based on the cut-off used in previous research.

## Results

### Sample characteristics

Taking the 32 participants as a whole, participants had a mean age of 28.0 years (*SD* = 8.8), a mean age of ED onset of 17.3 years (*SD* = 4.5), and a mean duration of illness of 10.7 years (*SD* = 8.9). Ninety-four percent of the participants were female. Eighty-two percent were single, 13% were married or in common-law relationships, and 6% were divorced. In terms of ethnic background, all participants identified themselves as Caucasian. Twenty-one participants (65.6%) were diagnosed with AN (9 with AN restricting subtype and 12 with AN binge-purge subtypes), 10 (31.3%) with BN, and 1 (3.1%) with Eating Disorder Not Otherwise Specified (EDNOS). The BMI ranges at admission to intensive treatment are as follows: AN-R (13.4-19.0), AN-BP (13.3-17.7), BN (16–26.2), EDNOS (21.22). Ninety-four percent of participants were currently receiving some form of additional treatment at pre-randomization. The most common forms of treatment were medications (most often anti-depressants), psychotherapy (with psychologists, psychiatrists, psychological associates, or social workers) and medical monitoring (with psychiatrists or general practitioners). Many participants were currently engaging in all three of these forms of treatment when they were randomized. Sixteen participants (50.0%) completed the intensive treatment program, whereas 16 (50.0%) dropped out prematurely.

There were no differences between the groups in terms of subtype, age, age of onset of their ED, length of illness, admission BMI, or type of intensive treatment program (inpatient vs. ambulatory treatment). Table 1 presents the sample characteristics broken down by treatment group.

**Table 1:** Sample characteristics by treatment group

**Measure**	**MI group (n = 16)**	**Control group (n = 16)**
Gender	15 Female	15 Female
	1 Male	1 Male
Age	M =28.0 years (S.D. = 9.5)	M = 28.4 years (S.D. = 7.8)
Ethnicity	16 Caucasian	16 Caucasian
Marital status	13 Single	13 Single
	2 Married/Common-law	2 Married
	1 Divorced	1 Divorced
Employment status	8 Students	6 Students
	6 Employed	7 Employed
	2 Unemployed	3 Unemployed
Age of onset	M = 17.1 years (S.D. = 4.0)	M = 17.5 years (S.D. = 5.1)
Duration of illness	M = 11.0 years (S.D. = 10.0)	M = 10.9 years (S.D. = 7.8)
Subtype	4 AN-R	5 AN-R
	8 AN-BP	5 AN-BP
	4 BN	6 BN
Admission BMI	*M* = 17.7 (*SD* = 2.9)	*M* = 18.4 (*SD* = 4.8)
Type of intensive treatment program	8 Inpatient program	7 Inpatient program
	8 Day hospital program	9 Day hospital program

There also were no significant differences between the groups on any of the questionnaires measured at pre-randomization. Table 2 presents means and SDs for all measures across time by treatment group, as well as within group effect sizes.

**Table 2:** Means and SDs for all measures across time by treatment group (n = 26)

**Measure and condition**	**Baseline**			**After pre-treatment**		
	**Mean**	**SD**	**Partial η**^**2**^	**Mean**	**SD**	**Partial η**^**2**^
PCED – pros subscale						
MI group	8.96	18.19	.001	1.60	23.41	.01
Control group	10.33	23.87		7.50	26.86	
PCED – cons subscale						
MI group	36.79	9.76	.001	40.27	9.23	.10
Control group	36.05	14.99		32.18	14.61	
MTC importance of eating						
MI group	8.09	2.74	.01	8.64	2.16	.000
Control group	7.60	2.23		8.60	1.80	
MTC confidence eating^a^						
MI group	6.09	2.12	.01	7.64	1.63	.04
Control group	5.57	2.38		6.86	2.07	
MTC readiness eating						
MI group	7.73	2.15	.16	8.73	2.15	.20
Control group	5.80	2.37		6.77	1.94	
MTC importance restriction						
MI group	8.18	2.27	.21	8.27	2.49	.04
Control group	5.53	2.90		7.47	2.31	
MTC confidence restriction						
MI group	6.05	2.49	.01	6.73	2.49	.01
Control group	6.47	2.31		7.27	2.31	
MTC readiness restriction						
MI group	6.80	1.79	.10	9.60	.89	.41
Control group	5.71	1.70		6.14	2.79	
MTC importance weight/shape						
MI group	9.00	1.00	.16	9.45	1.04	.14
Control group	7.47	2.23		8.33	1.68	
MTC confidence weight/shape						
MI group	6.73	1.95	.09	5.82	1.78	.04
Control group	5.00	3.21		6.67	2.19	
MTC readiness weight/shape^b^						
MI group	6.80	1.64	.06	9.80	.45	.29
Control group	5.71	2.63		7.29	2.69	

No analyses were significant at an α of 0.01.

^a^(n = 25).

^b^(n = 12).

### MI treatment integrity

According to the treatment integrity ratings, the quality of the MI sessions was high, with no session being rated as lower than 5 on either global dimension, and many receiving ratings of 6 or 7. The overall mean for the global “Empathy/Understanding” score was 5.88/7 (*SD* = .62), and the overall mean for the global “Spirit” score was 5.81/7 (*SD* = .75).

### The impact of MI on treatment completion rates

Intention to treat analysis, in which all participants are included regardless of whether they adhered to the treatment, was deemed to be inappropriate for the current study since it often underestimates the comparative effectiveness of a treatment [30]. The current study had several characteristics that contributed to that decision, including an emphasis on treatment integrity, a significant number of drop-outs and non-completers, and longer time between MI treatment and outcome. A logistic regression was run with condition (MI versus control) as the independent variable and treatment completion as the dependent variable. The overall model was significant (*Χ*^*2*^(1, 32) = 4.61, *p* = .032, Nagelkerke *R*^*2*^ = .18), and correctly predicted 69% of cases. Condition was found to be a significant predictor of outcome (*B* = 1.58, Wald Statistic = 4.27, *p* = .039), and examination of the odds ratio indicated that participants in the MI condition were 4.8 times more likely than those in the control condition to complete the subsequent treatment program. In this analysis, 11 (68.8%) of participants in the MI group completed the intensive treatment program, as compared to 5 (31.3%) of participants in the control group.

### Treatment condition and self-reported motivation

Twenty-six participants (81%) completed questionnaires at the second assessment. A series of repeated measures ANOVAs were conducted on each of the self-report measures, comparing participants’ scores at the second assessment to their scores at the first assessment. None of the interactions were significant at an α of 0.01; however, there were trends towards differences between the groups across time in the total score of the PCED Cons scale (*F* (1, 24) = 4.21, *p* = .05, partial η^2^ = .15), in that scores for the MI group increased over the four-week treatment period, whereas the same scores for the control group decreased over this time period. As well, there was a trend towards participants in the MI group having decreased confidence in their ability to stop being preoccupied with weight and shape, and the control group having increased confidence across time (*F* (1, 24) = 4.72, *p* = .04, partial η^2^ = .16). There was also a significant main effect for time: confidence in changing eating increased in both groups between filling out the first set of questionnaires and filling out the second (*F* (1, 23) = 12.97, *p* = .002, partial η^2^ = .36).

## Discussion

Eating disorders typically involve very low motivation to change and high rates of treatment drop out. The present study was a preliminary investigation of the impact of a brief MI intervention on subsequent completion rates of an intensive hospital treatment program for eating disorders. Findings indicated that patients who were randomly assigned to receive MI pre-treatment had a significantly higher rate of completion of the intensive treatment program compared to those who received treatment as usual. This was true despite the fact that the MI intervention was brief (three or four 50-minute sessions). These findings are among the first to empirical support for the utility of MI in improving later intensive treatment completion. They are consistent with the results of Treasure and colleagues [13], who found that motivational enhancement therapy was an effective first phase treatment for BN, and were in contrast to the results of Wade and colleagues [19], who did not find that a motivational interviewing pre-treatment led to improvements in subsequent treatment completion. The contrast between the intensive treatment completion rates of the two groups was impressive (i.e., 69% in the MI group vs. 31% in the control group). Odds ratio analysis revealed that participants in the MI condition were 4.8 times more likely to complete intensive treatment than were those in the control condition. Past research that has shown that completion of intensive hospital-based eating disorder treatment programs range from 32% to 51% [31,32]. The current study provides support for an association between an MI pre-treatment and increased likelihood of completing a subsequent intensive treatment program. Geller and colleagues recently concluded that MI may be of most benefit to improving the motivation levels of highly ambivalent patients with disordered eating [33], which may be similar to the population in this study. At the present time, we do not have any long-term follow-up data on the participants in the two groups. However, based on past research it is reasonable to assume that a large proportion of the participants who dropped out of the intensive treatment program will return for another admission of intensive treatment [34]. Thus, clinically speaking, there is a meaningful contrast between 31% of the MI group falling into this category of high risk for re-admission, as compared to 69% in the control group.

All of the participants in the current study were admitted into a well-established, intensive hospital-based treatment program for eating disorders where normalization of eating and weight are required. It would be interesting for future research to examine the impact of the type of program patients attend following MI. It may be that nature of the program and, more specifically, the degree to which there are behavioural change expectations, impacts on the apparent effectiveness of MI. Programs that require very high levels of behavioural change (including most inpatient and day hospital programs) may be those whose completion rates benefit most from MI pre-treatment.

### Treatment condition and measures of motivation

Although the current analysis determined that the MI intervention was associated with a higher probability of intensive treatment completion than no intervention, the mechanisms involved in this relationship are not yet clear. There were no significant differences found between the groups in terms of the measures of motivation across time, as had been predicted. An obvious limitation of the study was that the sample size was small, and it is possible that there was not enough statistical power to detect group differences on the self-reported measures of motivation. Also, since the majority of patients in both groups had started intensive treatment at the time that their motivation was assessed, their levels of motivation may have been equivalently heightened, which could explain why no significant differences between the treatment conditions were found. It is also important to consider that, according to the MI model; an individual’s level of motivation is constantly in flux. Some participants completed the measures on their own time and it is impossible to know what other factors may have been affecting them at the moment they sat down to fill out the measures. This limitation is inherent to some degree in all self-report measures, but may be particularly problematic when measuring motivation, given its potentially capricious nature. Future research examining changes in motivation through multiple assessments and in naturalistic environments (e.g., ecological momentary assessment approaches) and the triggers for these changes, would be especially useful to future research on motivation to change and eating disorders.

Another possibility is the measures of motivation in this study did not tap the important factors involved in change in an MI treatment. Amrhein and colleagues [35] investigated the relationship between client language in an MI session and outcome in treatment for drug use. They noted that, not only was there a distinction in client language between commitment (“I won’t be using.”), desire (“I want to quit doing drugs.”), ability (“I can do it…this is doable.”), need (“I need to stop.”), readiness (“I’m ready to do this.”), and reasons (“I’m killing myself.”) to change, but that commitment language was the only one of these categories to predict behavioural change. In addition, the remaining categories were found to predict commitment language. Using these categories, it appears that the measures of motivation used in the present study assessed participants’ levels of ability, need, readiness, and reasons to change, but not their actual commitment to changing. Although the results of the current study would suggest that these variables were equivalent in both groups, it is possible that the detailed exploration of these variables in MI may have led to more commitment language on the part of the MI participants, which then predicted increased probability of behavioural change (within the intensive treatment program). In other words, the effectiveness of MI may not lie in the client’s utterances of desire, ability, need, readiness, and reasons to change, but rather that making these utterances to a curious, non-judgmental therapist (who reflects these statements back to the client) may allow the client to synthesize these beliefs about change and may lead to statements of commitment to actually make a change, which would then lead to increased chances of completing a subsequent treatment program.

### Strengths of the current study

The present study had a number of strengths. First, it utilized a randomized controlled trial (RCT) methodology. RCTs are rare in research on ED treatment, and the present study represents an important first step in filling this deficiency in the literature. The present study was also particularly ecologically valid in that it had very few exclusion criteria: participants had to have symptoms that met criteria for a DSM-IV diagnosis of an ED (including EDNOS) and they had to have a minimum BMI of only 13. It was also important in terms of generalizability that the current study did not exclude participants who were acutely suicidal. It is interesting to note that two of the three participants in this study with known suicidal intent (including the person who was hospitalized) went on to complete the treatment program, which would suggest that individuals at risk for suicide are still amenable to treatment. Finally, treatment integrity was high; the study involved with close supervision of the MI sessions by a highly experienced clinician and the collection measures of theoretically related psychotherapeutic constructs.

### Limitations of the present study

Despite the above strengths, the current study also had a number of limitations. Importantly, participants in the “treatment as usual” control group did not receive individual therapist contact time equivalent to the MI group. Thus, it is impossible to know whether the increased likelihood of intensive treatment completion was associated with the unique components of MI specifically, or can be attributed to the opportunity for clients to simply talk with a supportive person before or during intensive treatment. Choosing and implementing an appropriate psychotherapeutic intervention that can serve as a control group against which to compare MI should be a goal for future, larger studies in this area. This was a naturalistic study and there were opportunities for concurrent treatments. There were also varying lengths of time between completion of the MI sessions and admission into intensive treatment. In fact, the majority of the completers were admitted to hospital prior to completing their MI sessions. This confound also influenced comparison scores between baseline and the second assessment of readiness. Participants in both conditions had access to other forms of treatment while they were waiting for intensive hospital treatment. Also, participants were not blind to their group assignment. As previous research has suggested that treatment expectations can affect the course and outcome of psychotherapy [36], it is possible that individuals in the treatment group had an increased likelihood of completing the intensive treatment program because they believed they received superior treatment than did those in the control group. The sample size of this pilot study was small. Despite the fact that significant group differences on treatment completion were found with the small sample, the reduced statistical power may have limited our ability to detect the psychological mechanisms through which MI treatment was related to completion of the subsequent intensive treatment program. Different measures of readiness for change might have yielded different results. And, finally, the current study was inclusive of participants who are often excluded from clinical trials, but was not able to control for all of the variables that the literature has suggested to be associated with outcome in intensive treatment for an eating disorder (e.g., low admission BMI, past treatment failures) [37].

## Conclusions

Despite the myriad risks associated with eating disorders, they are characterized by low motivation to change and high rates of treatment drop-out. Treatment drop-out has negative consequences from both the perspective of the well-being of the patient and from a health economics standpoint. The current study found that a brief, pre-treatment consisting of four individual sessions of MI was associated with a significantly increased likelihood of intensive treatment completion as compared to the control group. MI can be a useful intervention to engage individuals with severe eating disorders prior to participation in intensive treatment. MI as a brief prelude to hospital-based treatment for an eating disorder may help to improve completion rates in such programs. Further research is required to determine the precise therapeutic mechanisms of change in MI as well as to explore the impact of pre-treatment on intensive treatment completion more generally.

## Endnote

^a^Of potential note, this individual was eventually weight restored after attending this subsequent treatment program for the first time in her treatment history.

## Competing interests

The authors have no competing interests to declare.

## Authors’ contributions

CW conceived of the study, carried out the data collection, performed the statistical analysis, and wrote the majority of the manuscript. JM participated in the design and supervision of the study, and co-wrote the manuscript. HW participated in the design of the study, supervised some of the clinical intervention cases, and helped edit portions of the manuscript. JC participated in the design of the study, supervised some of the clinical intervention cases, and helped edit portions of the manuscript. This study was carried out as part of CW’s dissertation. All authors read and approved the final manuscript.
